# Precision-cut liver slices as an ex vivo model to evaluate antifibrotic therapies for liver fibrosis and cirrhosis

**DOI:** 10.1097/HC9.0000000000000558

**Published:** 2024-10-24

**Authors:** Yongtao Wang, Ben Leaker, Guoliang Qiao, Mozhdeh Sojoodi, Ibrahim Ragab Eissa, Eliana T. Epstein, Jonathan Eddy, Oizoshimoshiofu Dimowo, Georg M. Lauer, Motaz Qadan, Michael Lanuti, Raymond T. Chung, Bryan C. Fuchs, Kenneth K. Tanabe

**Affiliations:** 1 Division of Gastrointestinal and Oncologic Surgery, Massachusetts General Hospital and Harvard Medical School, Boston, Massachusetts, USA; 2 Liver Center, Division of Gastroenterology, Massachusetts General Hospital and Harvard Medical School, Boston, Massachusetts, USA; 3 Wellman Center for Photomedicine, Massachusetts General Hospital and Harvard Medical School, Boston, Massachusetts, USA; 4 Harvard-MIT program in Health Sciences and Technology, Massachusetts Institute of Technology, Boston, Massachusetts, USA; 5 Division of Thoracic Surgery, Massachusetts General Hospital and Harvard Medical School, Boston, Massachusetts, USA

**Keywords:** cirrhosis, EGFR inhibition, ex vivo model, liver fibrosis, precision-cut liver slices

## Abstract

**Background:**

Considering the lack of successful treatment options and poor prognosis for cirrhosis and cirrhosis-induced HCC, new platforms to investigate antifibrotic therapies are urgently needed. Precision-cut liver slice (PCLS) is a powerful ex vivo culture model that can supplement and potentially replace the traditional models.

**Methods:**

PCLS were prepared from 4 different murine cirrhotic models (choline-deficient, l-amino acid–defined, high-fat diet, thioacetamide, diethylnitrosamine, and carbon tetrachloride) and compared with in vivo murine experiments, in vitro hepatic stellate cells, and human cirrhotic PCLS.

**Results:**

PCLS viability in culture was stable for 72 hours. Treatment of erlotinib, an EGF receptor inhibitor, significantly inhibited profibrogenic gene expressions in PCLS from choline-deficient, l-amino acid–defined, high-fat diet or thioacetamide-induced cirrhotic rats. Erlotinib treatment of PCLS from diethylnitrosamine or carbon tetrachloride–induced cirrhotic rats inhibited the expression of profibrogenic genes, which was consistent with the impact of erlotinib on these genes in in vivo diethylnitrosamine or carbon tetrachloride–induced cirrhosis. In addition, in hepatic stellate cells at PCLS from normal mice, erlotinib treatment inhibited TGF-β1–upregulated expression of *Acta2*. Similar expression results were observed in in vitro hepatic stellate cells. Expression of key regulators of fibrosis progression and regression were also significantly altered. Changes in profibrogenic gene expression under erlotinib treatment were also corroborated with human cirrhotic PCLS.

**Conclusions:**

Responses to antifibrotic interventions can be detected and quantified with PCLS at the gene expression level. The antifibrotic effects of erlotinib are consistent between PCLS models of murine cirrhosis and those observed in vivo and in vitro. These results were verified in human cirrhotic PCLS. PCLS is an excellent model for assessing antifibrotic therapies that are aligned with the principles of replacement, reduction, and refinement (3Rs), and it will benefit preclinical and clinical research for human fibrosis and cirrhosis.

## Graphical abstract

**Figure:**
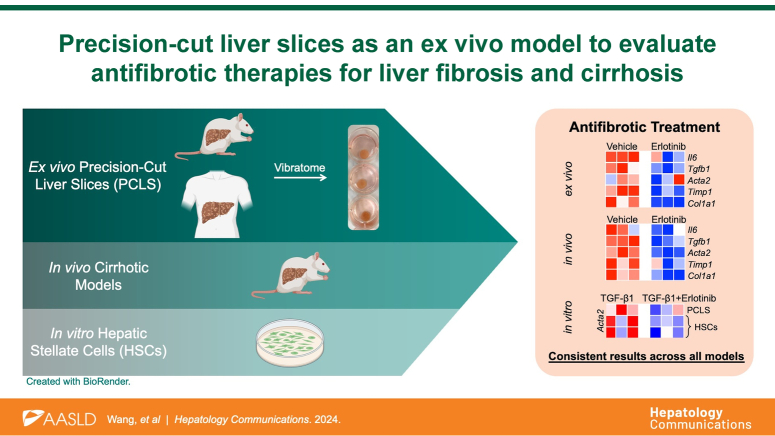

## INTRODUCTION

Cirrhosis is the final stage of liver fibrosis caused by many forms of liver injury, such as chronic hepatitis B and C, chronic alcohol use disorder, and metabolic dysfunction–associated steatohepatitis (previously known as nonalcoholic steatohepatitis and NASH).^1^,^2^ In 2015, cirrhosis affected 2.8 million people and resulted in 1.3 million deaths globally.^3^,^4^ Cirrhosis also contributes to the vast majority of HCC cases, which is the sixth most common cancer worldwide and the second leading cause of cancer-related death.^5^ Considering the lack of successful treatment options and poor prognosis for cirrhosis,^6^ new strategies and platforms to investigate antifibrotic therapies are urgently needed.

Many of the limitations of cellular and animal models of liver fibrosis can be overcome by using the relatively recently proposed precision-cut liver slice (PCLS) model. This technique maintains thin slices of liver tissue in culture for several days, enabling drugs to be tested in an ex vivo setting that retains the architecture and cell populations of the liver. Compared to 2-dimensional in vitro cell culture, PCLS better models the interactions and signal transduction pathways between different cell types.^7^,^8^ The PCLS model also offers the benefit of reduced animal experiments, which can be expensive and time-consuming, and is in line with the principles of replacement, reduction, and refinement (3Rs).^9^

PCLS has been used to study various liver conditions, such as normal liver function,^10^ drug metabolism,^11^ DILI and toxicity,^12^,^13^,^14^ fatty liver,^15^ early stages of liver fibrosis,^8^,^15^,^16^,^17^,^18^,^19^,^20^ and immunological responses.^21^ A study using PCLS derived from rats subjected to bile duct ligation supported PCLS ability to perform a molecular antifibrotic evaluation with transcriptomic characterization.^22^ However, PCLS as a platform to evaluate antifibrotic therapies for cirrhosis, a more severe stage of liver disease, has not been reported.

Previous experiments from our lab have demonstrated the efficacy of a small-molecule EGF receptor inhibitor, erlotinib, as an antifibrotic agent in murine models of liver fibrosis.^23^ Erlotinib inhibited the activation of hepatic stellate cells (HSCs), stopped the progression of cirrhosis, and prevented subsequent development of HCC. Here, erlotinib was used as an example of antifibrotic therapy in PCLS. The aim of this study was to assess whether responses to antifibrotic interventions can be detected and quantified with PCLS.

## METHODS

### Chemicals

Stock solution of 50 mM erlotinib (Tarceva, Genentech) was prepared in DMSO.

### Animal models for PCLS

All animal experiments were approved by the MGH Institutional Animal Care and Use Committee (IACUC). PCLS were prepared from 4 established murine models of cirrhosis: choline-deficient, l-amino acid–defined, high-fat diet (CDAHFD), thioacetamide (TAA), diethylnitrosamine (DEN), and carbon tetrachloride (CCl_4_).

The DEN model of cirrhosis was prepared as described.^23^ Male Wistar rats received weekly i.p. injections of DEN (Sigma) at 50 mg/kg for 18 weeks. The CCl_4_ model of cirrhosis was prepared as described.^23^ Male C57Bl/6 mice were treated 3 times a week for 18 weeks with 0.1 mL of 40% CCl_4_ (Sigma) in olive oil by oral gavage. The CDAHFD model of cirrhosis was induced as described.^24^ Male C57Bl/6 mice received CDAHFD (l-amino acid diet with 60 kcal% fat with 0.1% methionine and no added choline; Research Diets Inc. A06071302) for 16 weeks. The TAA model of cirrhosis was induced as described.^25^ Male Wistar rats received i.p. injections with 200 mg/kg TAA (Sigma) twice a week for 12 weeks.

### PCLS as an ex vivo model of liver biology

PCLS from healthy or cirrhotic livers were prepared as described.^7^,^26^,^27^ Tissue samples were glued to the mounting stage of a 7000smz-2 vibratome instrument (Campden Instruments Limited) and submersed in sterile Krebs Henseleit buffer (Sigma-Aldrich). Using 7550-1-C ceramic blades (Campden Instruments Limited), the tissue was cut into 250 μm slices at an advance speed of 0.1 mm/s, with 2.5 mm amplitude and 50 Hz frequency. Slices from human and rat livers were trimmed to a uniform size with an 8 mm biopsy punch (Acuderm Inc.), and slices from mouse livers were trimmed with a 6 mm biopsy punch. PCLS were transferred into 8 μm-pore Transwell inserts and cultured in standard 6-well plates (Corning), with 2 mL William’s E medium (Sigma-Aldrich) containing 2.0 g/L glucose, 10% fetal bovine serum (Gibco), 2 mM l-glutamine supplement (Gibco), 100 U/mL penicillin, and 100 μg/mL streptomycin (Lonza), at 37°C in a humidified atmosphere of 5% CO_2_.

PCLS from cirrhotic animals were then treated with 5 μM erlotinib or vehicle control for 72 hours. PCLS from normal murine livers were treated with 10 ng/mL TGF-β1 for 24 hours, then cotreated with 10 ng/mL TGF-β1 and 5 μM erlotinib for an additional 72 hours.

### In vivo erlotinib experiments

As described,^23^ male Wistar rats received weekly i.p. injections of 50 mg/kg DEN for 18 weeks. Starting from week 8, rats received daily i.p. injections with either 2 mg/kg erlotinib or vehicle control (n = 8 per group). All rats were sacrificed at week 19 after a 1-week washout period to eliminate the acute effects of DEN. The livers from this previous in vivo experiment were used in this current study.

As described,^23^ male A/J mice were treated 3 times a week for 18 weeks with 0.1 mL of 40% CCl_4_ in olive oil by oral gavage. Mice received daily i.p. injections of control or 2 mg/kg erlotinib from weeks 13 to 19 (n = 8 per group). Mice were sacrificed at week 19 after a 1-week washout to eliminate the acute effects of CCl_4_. The livers from this previous in vivo experiment were used in this current study.

### Cell culture

Culturing of human HSCs was performed as described.^28^,^29^ LX2 was obtained from Dr Raymond Chung, who received the cells from Dr Scott Friedman. TWNT4 was obtained from Dr Sangeeta Bhatia. Cells were grown in DMEM (Cellgro) or Roswell Park Memorial Institute (RPMI) 1640 medium (HyClone) containing 15% fetal bovine serum (Gibco), 1 mM sodium pyruvate (Gibco), 100 U/mL penicillin sodium, and 100 μg/mL streptomycin sulfate (Lonza), at 37°C in a humidified atmosphere of 5% CO_2_.

### MTS

3-(4,5-dimethylthiazol-2-yl)-5-(3-carboxymethoxyphenyl)-2-(4 sulfophenyl)-2H-tetrazolium assay was performed as described.^21^,^30^,^31^ Individual PCLS were placed in a 24-well plate with 400 μL William’s E medium and 80 μL 3-(4,5-dimethylthiazol-2-yl)-5-(3-carboxymethoxyphenyl)-2-(4 sulfophenyl)-2H-tetrazolium solution (Abcam) for each well. Plates were incubated at 37°C in standard culture conditions for 1 hour, then mixed briefly on a shaker. Supernatants were transferred to a 96-well plate, and absorbance was measured using a plate reader (Molecular Devices) at OD = 490 nm.

### Histological hematoxylin and eosin and Sirius red staining

Initially, PCLS were fixed in formalin for 3 days at room temperature, then paraffin-embedded and sectioned into 7 μm slices for hematoxylin and eosin (H&E) and Sirius red staining with standard protocols.^32^,^33^ However, we found that PCLS from cirrhotic livers were fragile and easily degraded during sectioning and Sirius red staining (Supplemental Figure S3A, left panel, http://links.lww.com/HC9/B65). The standard protocol was then modified. PCLS were fixed in formalin at 4^o^C overnight, embedded in paraffin, and then sectioned into 10 μm slices. The time for all steps was also reduced to half of the standard time. This modified protocol resulted in higher-quality stains and retained the structure of the PCLS (Supplemental Figure S3A, right panel, http://links.lww.com/HC9/B65). The amount of collagen in Sirius red–stained sections was scored according to the modified Ishak method^34^ as described in Supplemental Table S1, http://links.lww.com/HC9/B65. The collagen proportionate area was morphometrically quantified with image processing software (ImageJ, NIH).

### Quantitative RT-PCR

Quantitative reverse transcription polymerase chain reaction was performed as described.^35^,^36^,^37^ Total RNA was isolated from rat liver tissues using TRIzol (Invitrogen) and subsequently treated with DNase I (Promega) according to the manufacturer’s instructions. One microgram of total RNA from each sample was used to synthesize cDNA (SuperScript III First-Strand Synthesis SuperMix for quantitative reverse transcription polymerase chain reaction, Invitrogen). Quantitative real-time PCR was performed using the 7900HT Fast Real-Time PCR System (Thermo Fisher) with commercial TaqMan primers (Thermo Fisher), as shown in Supplemental Table S2, http://links.lww.com/HC9/B65.

### Human samples

All human samples were obtained in accordance with the protocols approved by the Mass General Brigham Institutional Review Boards (IRBs).

### Statistical analysis

All values were expressed as mean ± SEM. Two-tailed Student *t* tests were performed to compare data between the control and 1 experimental group, and one-way ANOVA followed by post hoc Tukey tests with 2-tailed distribution were performed to analyze data among groups of 3 or more. Graphs were prepared with GraphPad Prism v6.0c software. All experiments were independently repeated 3 times. Significance is represented by *p < 0.05, **p < 0.01, ***p < 0.001 versus control, ns indicates not significant. The heatmaps were prepared with 3 independently repeated experiments by Morpheus (https://software.broadinstitute.org/morpheus).

## RESULTS

### Stable viability of PCLS

**FIGURE 1:**
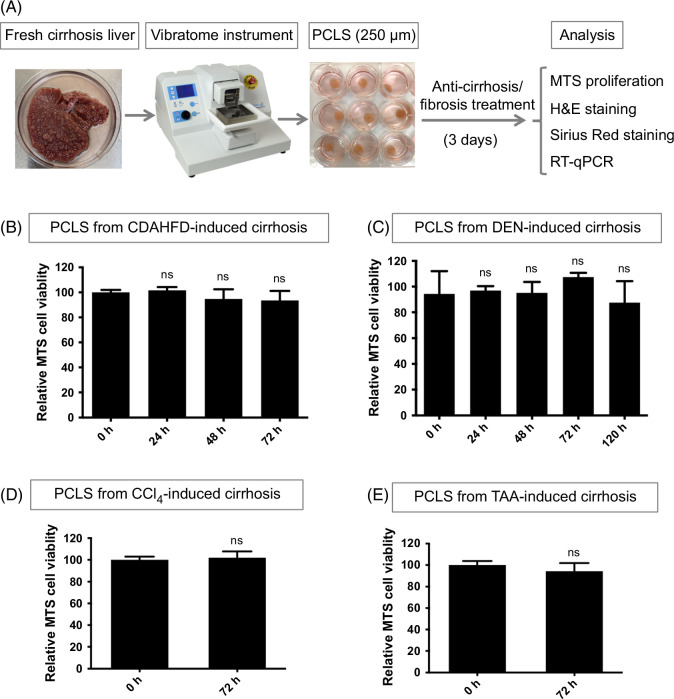
Evaluation of viability in PCLS. (A) Workflow for PCLS preparation and treatment. Viability analyzed with MTS assays for PCLS from (B) CDAHFD, (C) DEN, (D) CCl_4_, and (E) TAA-induced cirrhosis rats. Experiments were independently repeated 3 times (n = 3). All values were expressed as the mean ± SEM with 2-tailed Student *t* tests or 1-way ANOVA analysis; ns indicates not significant. All treatment groups were statistically compared with the mock group. Abbreviations: CCl_4_, carbon tetrachloride; CDAHFD, choline-deficient, l-amino acid–defined, high-fat diet; DEN, diethylnitrosamine; H&E, hematoxylin and eosin; MTS, 3-(4,5-dimethylthiazol-2-yl)-5-(3-carboxymethoxyphenyl)-2-(4 sulfophenyl)-2H-tetrazolium; PCLS, precision-cut liver slices; RT-qPCR, reverse transcription quantitative polymerase chain reaction; TAA, thioacetamide.

PCLS with 250 μm thickness were prepared and further cultured with treatment (Figure 1A, Supplemental Figure S1, http://links.lww.com/HC9/B65). To ensure minimal tissue degradation ex vivo, the period of stable viability was determined. 3-(4,5-dimethylthiazol-2-yl)-5-(3-carboxymethoxyphenyl)-2-(4 sulfophenyl)-2H-tetrazolium assays showed the viability of PCLS from mouse CDAHFD-induced, rat DEN-induced, rat TAA-induced, and mouse CCl_4_-induced established cirrhosis were not significantly changed after 72 hours in culture (Figures 1B–E, Supplemental Figures S2A, B, http://links.lww.com/HC9/B65). This time point was used for the remaining experiments with cirrhotic PCLS. The DEN model was also used to investigate the lifespan of cirrhotic PCLS. The nominal decrease in viability after 5 days was not statistically significant (Figure 1C). The CDAHFD, TAA, and DEN models were further used to determine whether erlotinib had an impact on PCLS viability. There was no difference between vehicle and erlotinib-treated slices with these models at 72 hours (Supplemental Figures S2C–E, http://links.lww.com/HC9/B65).

### Antifibrotic treatment of PCLS from CDAHFD-induced mouse cirrhosis and TAA-induced rat cirrhosis

**FIGURE 2:**
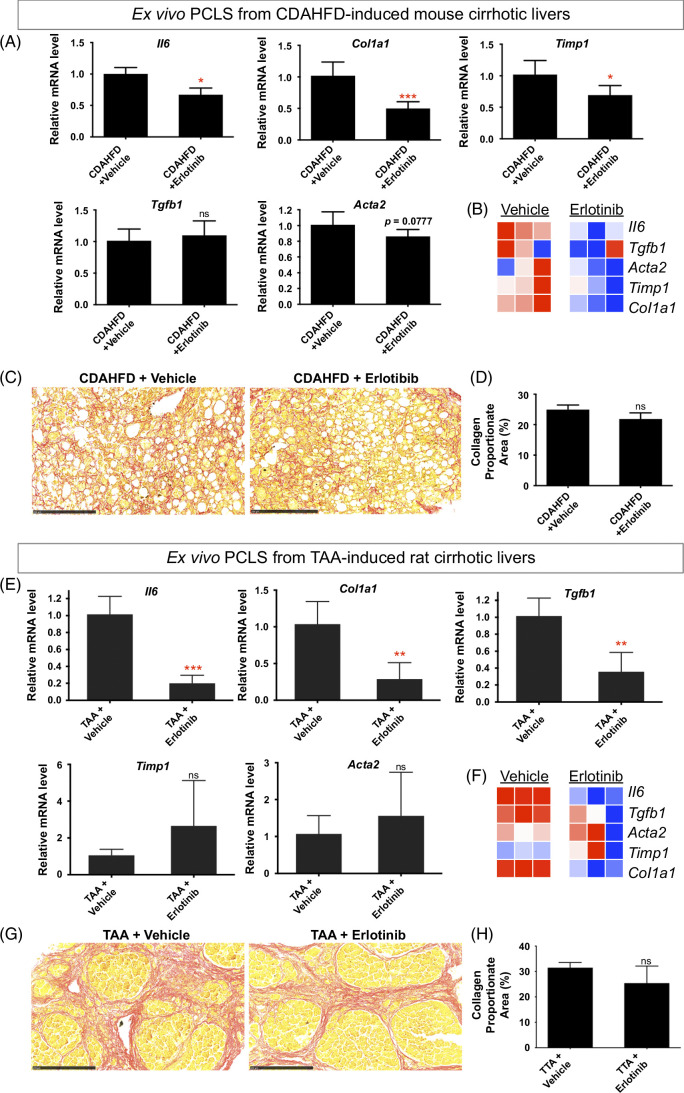
Antifibrotic evaluation in PCLS from CDAHFD-induced mouse cirrhosis and TAA-induced rat cirrhosis. (A) Quantitative RT-PCR analysis of profibrogenic genes. (B) Heatmap of profibrogenic genes from 3 independently repeated experiments. Amount of collagen (C) measured with Sirius red staining and (D) quantified with collagen proportionate area analysis in PCLS from CDAHFD-induced mouse cirrhosis after 5 μM erlotinib treatment for 72 hours. (E) Quantitative RT-PCR analysis of profibrogenic genes. (F) Heatmap of profibrogenic genes from 3 independently repeated experiments. Amount of collagen (G) measured with Sirius red staining and (H) quantified in PCLS from TAA-induced rat cirrhosis after 5 μM erlotinib treatment for 72 hours. The images of Sirius red staining for both CDAHFD and TAA models were presented at ×20 magnification. Experiments were independently repeated 3 times (n = 4). All values were expressed as the mean ± SEM with 2-tailed Student’s *t* tests. Significance is represented by *p < 0.05, **p < 0.01, ***p < 0.001, ns indicates not significant. Abbreviations: CDAHFD, choline-deficient, l-amino acid–defined, high-fat diet; PCLS, precision-cut liver slices; RT-PCR, reverse transcription polymerase chain reaction; TAA, thioacetamide.

To first assess whether responses to antifibrotic interventions can be detected and quantified with PCLS, PCLS from CDAHFD-induced and TAA-induced cirrhosis were used. CDAHFD-induced cirrhosis^32^ was confirmed with Sirius red staining (Supplemental Figure S4A, http://links.lww.com/HC9/B65). Erlotinib treatment of PCLS from CDAHFD-induced mouse cirrhotic livers for 72 hours significantly suppressed the expression of the profibrogenic genes *Il6*, *Col1a1*, and *Timp1* (Figures 2A, B) and suppressed *Acta2* (also named *αSMA*) expression with marginal significance (*p* = 0.0777). No significant effect was observed on the expression of *Tgfb1*. This short-term exposure of PCLS slices to erlotinib did not significantly reduce the amount of collagen measured with Sirius red staining (Figures 2C, D). No change was evident in H&E-stained morphology (Supplemental Figure S4B, http://links.lww.com/HC9/B65).

Cirrhosis was induced by TAA^38^ and confirmed with Sirius staining (Supplemental Figure S5A, http://links.lww.com/HC9/B65). Erlotinib treatment of PCLS from TAA-induced rat cirrhotic livers for 72 hours significantly inhibited the expression of the profibrogenic genes *Il6*, *Tgfb1*, and *Col1a1* (Figures 2E, F). No significant changes in the expression of *Timp1* and *Acta2* were observed. This short-term exposure to erlotinib did not significantly reduce the amount of collagen (Figures 2G, H). No change was evident on H&E staining (Supplemental Figure S5B, http://links.lww.com/HC9/B65).

Taken together with the CDAHFD and TAA data above, acute responses to antifibrotic interventions can be detected and quantified with PCLS at the gene expression level.

### Antifibrotic treatment in PCLS from DEN-induced rat cirrhosis compared to in vivo DEN cirrhosis model

**FIGURE 3:**
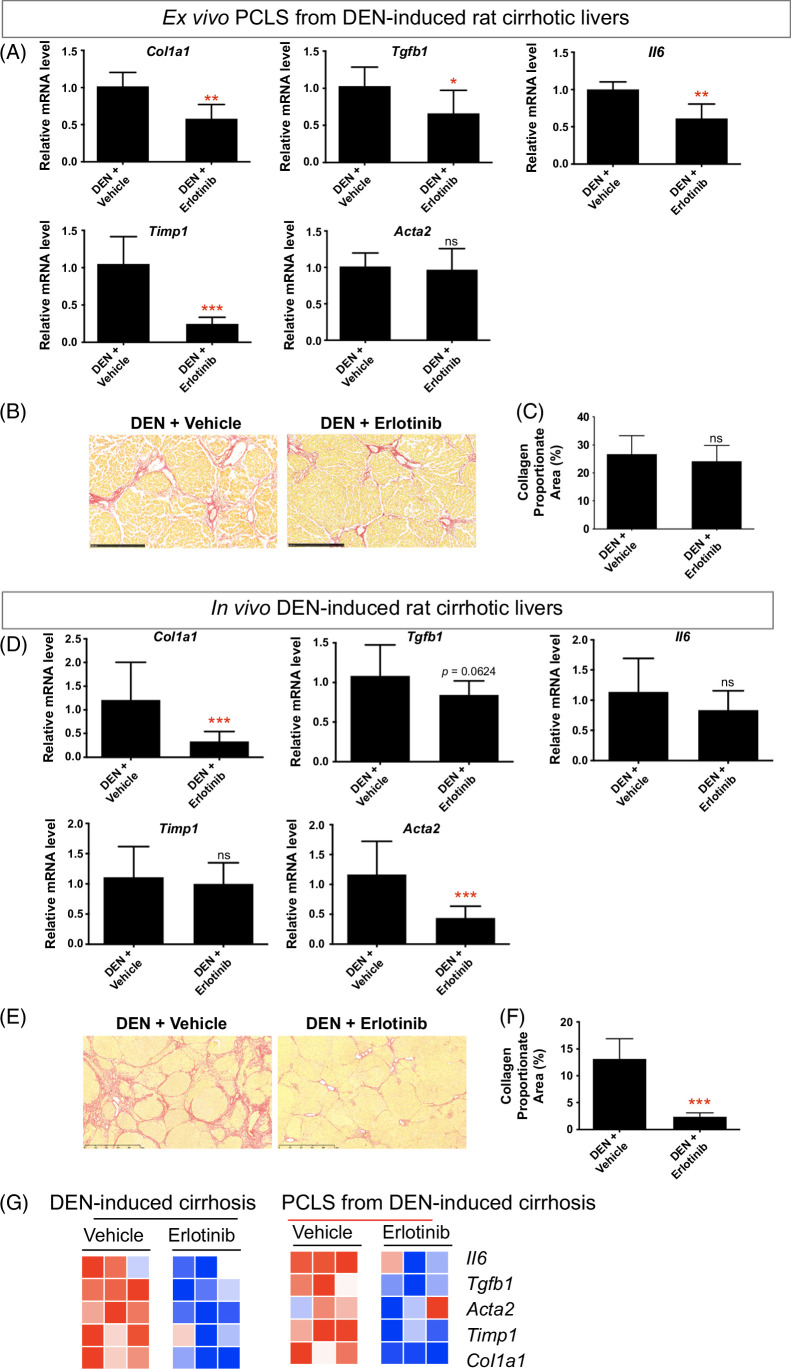
Comparison of antifibrotic therapy in ex vivo PCLS from DEN-induced *rat* cirrhosis with in vivo DEN cirrhotic model. For ex vivo PCLS analysis, (A) quantitative RT-PCR analysis of profibrogenic genes. Amount of collagen (B) measured with Sirius red staining and (C) quantified with collagen proportionate area analysis in PCLS after 5 μM erlotinib treatment for 72 hours. Experiments were independently repeated 3 times (n = 4). For in vivo analysis, (D) quantitative RT-PCR analysis of profibrogenic genes. Amount of collagen (E) measured with Sirius red staining and (F) quantified after daily i.p. injections of 2 mg/kg erlotinib for 10 weeks. (G) Comparison of heatmaps of profibrogenic genes for corresponding models. All values were expressed as the mean ± SEM with 2-tailed Student *t* tests. Significance is represented by *p < 0.05, **p < 0.01, ***p < 0.001, ns indicates not significant. Abbreviations: DEN, diethylnitrosamine; PCLS, precision-cut liver slices; RT-qPCR, reverse transcription quantitative polymerase chain reaction.

DEN-induced cirrhosis^23^,^39^,^40^,^41^,^42^ was confirmed with Sirius staining (Supplemental Figure S6A, http://links.lww.com/HC9/B65). Exposure of PCLS from DEN-induced rat cirrhotic liver to erlotinib for 72 hours significantly suppressed the expression of the profibrogenic genes *Col1a1*, *Tgfb1*, *Il6*, and *Timp1* (Figure 3A). No significant difference in the amount of collagen (Figures 3B, C) and H&E staining (Supplemental Figure S6B, http://links.lww.com/HC9/B65) were observed after erlotinib treatment of PCLS slices for 72 hours.

To compare the effect of erlotinib between ex vivo cirrhotic PCLS and an in vivo cirrhosis model, we analyzed livers obtained from DEN-induced cirrhotic rats^23^ that had been treated with erlotinib for 10 weeks (Supplemental Figure S6C, http://links.lww.com/HC9/B65). As observed in the short-term PCLS model, long-term treatment of DEN-induced cirrhotic rats resulted in a significant reduction in liver *Col1a1* and *Acta2* expression (Figure 3D) and marginally reduced the expression of *Tgfb1* (*p* = 0.0624). No effect on the expression of *Il6* and *Timp1* was observed. As previously published, 10 weeks of erlotinib treatment in these rats also significantly reduced the amount of collagen (Figures 3E, F).

The heatmap showed that expression of profibrogenic genes was inhibited after erlotinib treatment both in PCLS from DEN-induced cirrhotic rats and in in vivo DEN-induced cirrhotic rats (Figure 3G), indicating that the ex vivo PCLS from DEN-induced cirrhotic rats was demonstrated to closely resemble the molecular features after erlotinib treatment observed in in vivo DEN-induced rat cirrhosis.

### Antifibrotic treatment in PCLS from CCl_4_-induced mouse cirrhosis compared to in vivo CCl_4_ cirrhosis model

**FIGURE 4:**
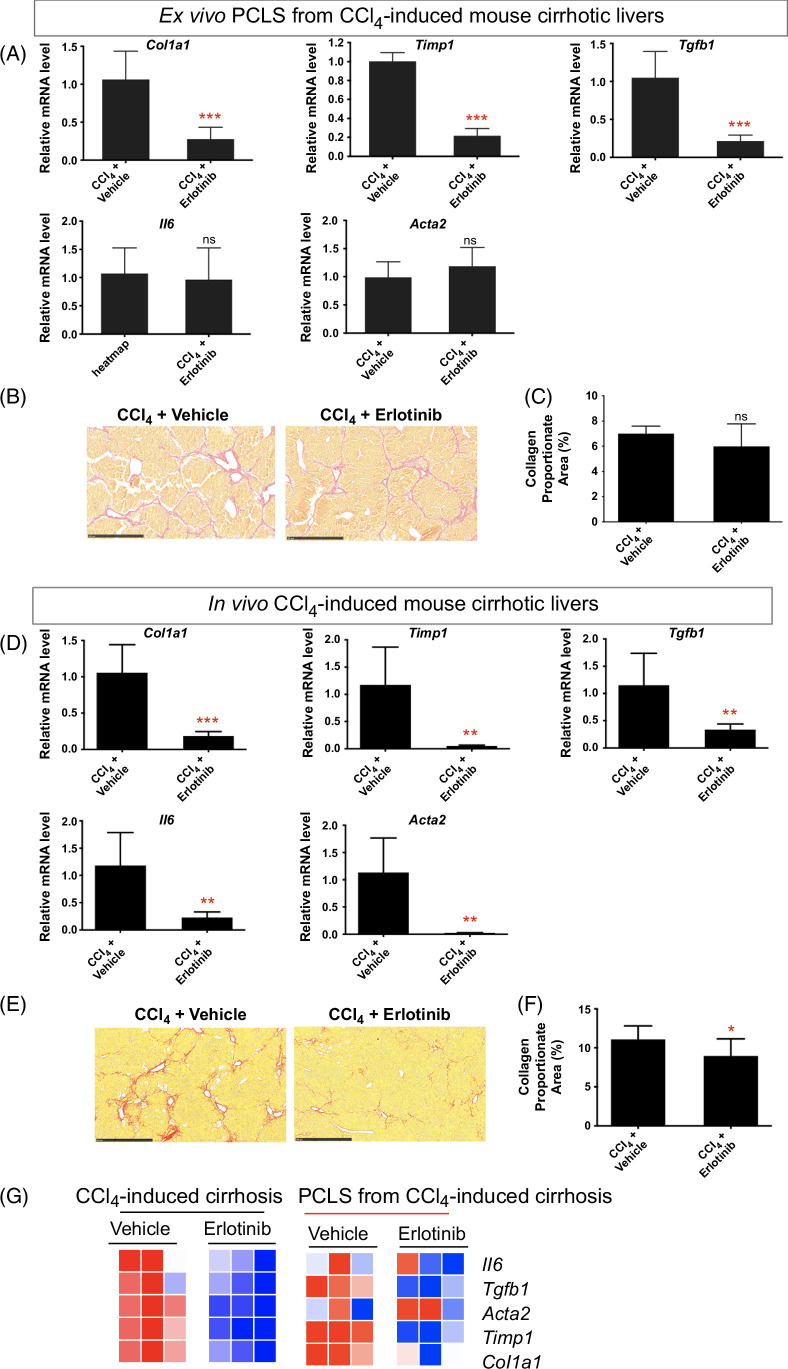
Comparison of antifibrotic therapy in ex vivo PCLS from CCl_4_-induced mouse cirrhosis with in vivo CCl_4_ cirrhotic model. For ex vivo PCLS analysis, (A) quantitative RT-PCR analysis of profibrogenic genes. Amount of collagen (B) measured with Sirius red staining and (C) quantified with collagen proportionate area analysis in PCLS after 5 μM erlotinib treatment for 72 hours. Experiments were independently repeated 3 times (n = 4). For in vivo analysis, (D) quantitative RT-PCR analysis of profibrogenic genes. Amount of collagen (E) measured with Sirius red staining and (F) quantified after daily i.p. injections of 2 mg/kg erlotinib for 6 weeks. (G) Comparison of heatmaps of profibrogenic genes for corresponding models. All values were expressed as the mean ± SEM with 2-tailed Student’s *t* tests. Significance is represented by *p < 0.05, **p < 0.01, ***p < 0.001, ns indicates not significant. Abbreviations: CCl_4_, carbon tetrachloride; PCLS, precision-cut liver slices; RT-qPCR, reverse transcription quantitative polymerase chain reaction.

CCl_4_-induced cirrhosis^23^ was confirmed with Sirius red staining (Supplemental Figure S7A, http://links.lww.com/HC9/B65). Short-term exposure of PCLS from CCl_4_-induced mouse cirrhotic liver to erlotinib significantly suppressed the expression of the profibrogenic genes *Timp1*, *Col1a1*, and *Tgfb1* (Figure 4A) but did not affect the expression of *Il6* and *Acta2*. No significant difference in the amount of collagen (Figures 4B, C) and H&E staining (Supplemental Figure S7B, http://links.lww.com/HC9/B65) was observed after erlotinib treatment of the PCLS for 72 hours.

To compare the effect of erlotinib between ex vivo cirrhotic PCLS and an in vivo cirrhosis model, we analyzed liver obtained from CCl_4_-induced cirrhotic mice^23^ that had been treated with erlotinib for 6 weeks (Supplemental Figure S7C, http://links.lww.com/HC9/B65). Six weeks of erlotinib treatment in these mice significantly suppressed the expression of *Timp1*, *Col1a1*, *Tgfb1*, *Il6*, and *Acta2* in the liver (Figure 4D) and reduced the amount of collagen (Figures 4E, F).

The heatmap showed that similar decreased expression of the profibrogenic genes in the CCl_4_‐treated mouse livers was observed in PCLS from CCl_4_-induced cirrhotic mice after erlotinib treatment (Figure 4G), suggesting that the ex vivo PCLS model indeed recapitulates antifibrotic effect observed in in vivo mouse cirrhosis. Taken together with the DEN and CCl_4_ data above, these results demonstrate that erlotinib induces acute effects in PCLS that are similar to those observed in vivo in these 2 murine models of cirrhosis.

### Antifibrotic treatment in HSCs from PCLS compared to in vitro HSCs

**FIGURE 5:**
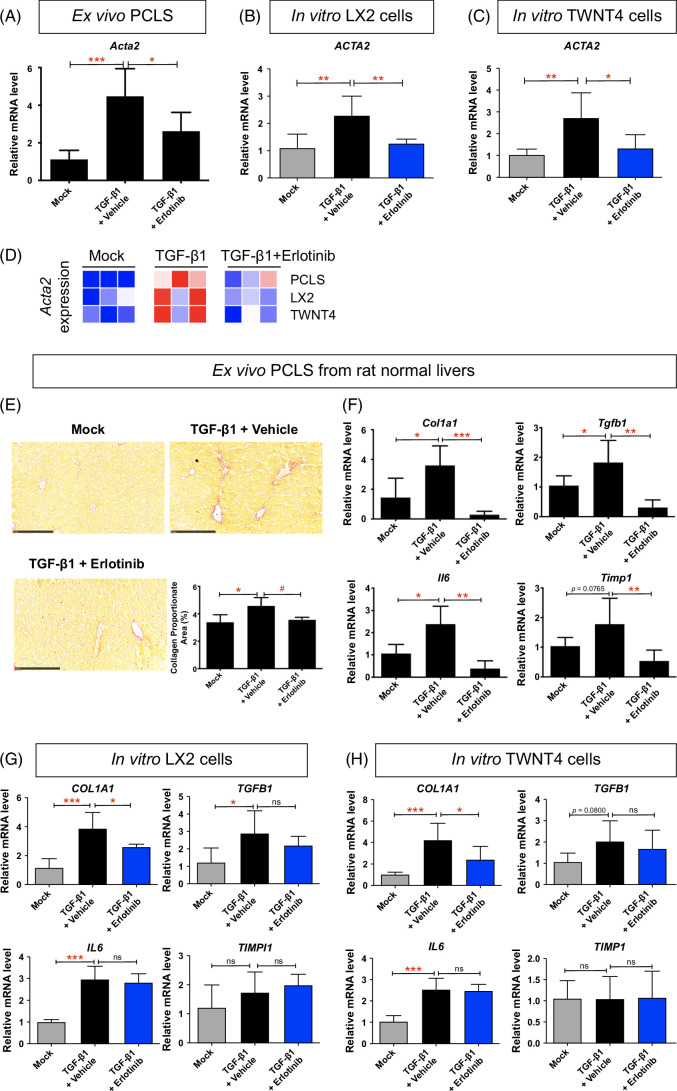
Comparison of antifibrotic evaluation in ex vivo HSCs from PCLS with in vitro HSCs. Quantitative RT-PCR analysis of *Acta2* mRNA from (A) PCLS from normal rats, (B) LX2, and (C) TWNT4 cells. PCLS or HSCs were activated with 10 ng/mL TGF-β1 for 24 hours and further treated with 5 μM erlotinib for 72 hours. (D) Heatmap of *Acta2* expression in corresponding models. (E) Amount of collagen measured with Sirius red staining and quantified with collagen proportionate area analysis in PCLS from normal rats. Quantitative RT-PCR analysis of *Il6*, *Tgfb1*, *Timp1*, and *Col1a1* mRNA from (F) PCLS from normal rats, (G) LX2, and (H) TWNT4 cells. These experiments were repeated 3 times (n = 4). All values were expressed as the mean ± SEM with 1-way ANOVA analysis. Significance is represented by *p < 0.05, **p < 0.01, ***p < 0.001, ns indicates not significant. Abbreviations: PCLS, precision-cut liver slices; RT-qPCR, reverse transcription quantitative polymerase chain reaction.

In PCLS from normal rats (Supplemental Figure S8A, http://links.lww.com/HC9/B65), HSCs identified by the expression of *Acta2* represented only a small minority of cells.^43^ TGF-β1 treatment for 72 hours induced the expression of *Acta2* in HSCs of PCLS, while treatment with erlotinib significantly inhibited the expression of *Acta2* (Figure 5A). In stellate cell lines LX2 (Figure 5B) and TWNT4 (Figure 5C), TGF-β1 significantly induced the expression of *ACTA2*, while treatment with erlotinib for 72 hours suppressed *ACTA2* expression. The heatmap also supported that, in HSCs at PCLS from normal mice, erlotinib treatment inhibited TGF-β1–upregulated expression of *Acta2*. Similar expression results were observed in in vitro HSCs (Figure 5D).

Erlotinib treatment for 72 hours also reduced TGF-β1–induced collagen deposition (Figure 5E) on the morphological level. These results demonstrate that erlotinib induces similar effects in HSCs in PCLS and in vitro HSCs.

TGF-β1 treatment also induced the expression of other profibrogenic genes *Il6*, *Tgfb1 Timp1*, and *Col1a1* in PCLS, while treatment with erlotinib significantly inhibited the expression of these genes (Figure 5F, Supplemental Figure S8B, http://links.lww.com/HC9/B65). In stellate cell lines LX2 (Figure 5G, Supplemental Figure S8B, http://links.lww.com/HC9/B65) and TWNT4 (Figure 5H, Supplemental Figure S8BS8B, http://links.lww.com/HC9/B65), TGF-β1 significantly induced the expression of *COL1A1*, while treatment with erlotinib suppressed *COL1A* expression. However, *TGFB1* and *IL6* did not respond to erlotinib treatment, and *TIMP1* did not respond to TGF-β1 and erlotinib treatment on these cells.

### Effect of antifibrotic therapy on matrix metalloproteinases and TIMPs expression in cirrhotic PCLS

**FIGURE 6:**
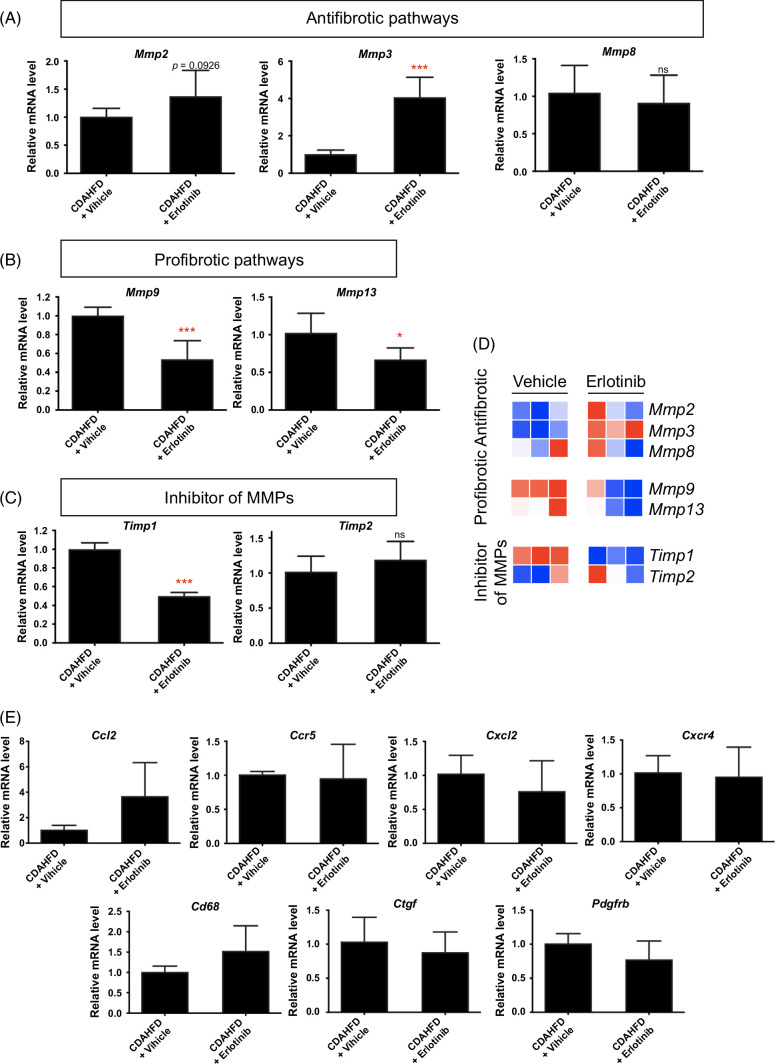
Regulatory pathways of the antifibrotic evaluation of erlotinib in cirrhotic PCLS. Quantitative RT-PCR analysis of the mRNA expression on (A) antifibrotic pathways, (B) profibrotic pathways, and (C) inhibitory pathways of MMPs in PCLS from CDAHFD-induced cirrhosis mice after erlotinib treatment for 72 hours. (D) These experiments were repeated 3 times (n = 4) and summarized in the form of a heatmap. (E) Quantitative RT-PCR analysis on additional regulatory genes for fibrosis generation and degradation. All values were expressed as the mean ± SEM with 2-tailed Student *t* tests. Significance is represented by *p < 0.05, ***p < 0.001, ns indicates not significant. Abbreviations: CDAHFD, choline-deficient, l-amino acid–defined, high-fat diet; MMP, matrix metalloproteinase; PCLS, precision-cut liver slices; RT-PCR, reverse transcription quantitative polymerase chain reaction.

To explore other potential mechanisms of antifibrotic therapy of erlotinib in cirrhotic PCLS, we assessed the expression of other regulatory pathways for fibrosis generation and degradation. In livers from CDAHFD-induced cirrhotic mice, exposure of PCLS slices to erlotinib for 72 hours significantly increased the expression of *Mmp2*, *Mmp3*, and *Mmp8* (Figure 6A), indicating activation of antifibrotic pathways. Erlotinib treatment also significantly decreased *Mmp9*, *Mmp13* (Figure 6B), and *Timp1* (Figure 6C) expression, reflecting suppression of profibrotic pathways. Decreased *Timp1* expression was also observed in PCLS from CDAHFD (Figure 2A), DEN (Figure 3A), and CCl_4_-induced (Figure 4A) cirrhotic livers. These observations were summarized in the form of a heatmap (Figure 6D). Additional regulatory genes like C-C motif ligand 2 (Ccl2), C-C motif chemokine receptor 5 (Ccr5), C-X-C motif chemokine ligand 2 (Cxcl2), C-X-C motif chemokine receptor 4 (Cxcr4), Cd68, connective tissue growth factor (Ctgf), and platelet-derived growth factor receptor beta (Pdgfrb) were analyzed.^23^ No significant changes in these genes were observed (Figure 6E).

### Antifibrotic evaluation in human cirrhotic PCLS

**FIGURE 7:**
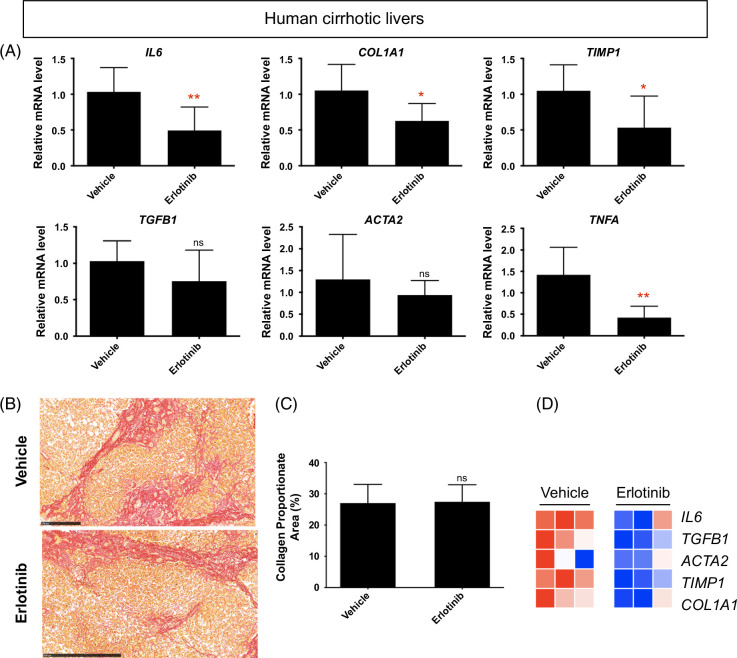
The antifibrotic evaluation in human cirrhotic PCLS. (A) Quantitative RT-PCR analysis of mRNA expression of profibrogenic genes. Amount of collagen (B) measured with Sirius red staining and (C) quantified in PCLS from human cirrhosis treated with 5 μM erlotinib for 72 hours. (D) Heatmap of profibrogenic genes from 3 independently repeated experiments (n = 4). All values were expressed as the mean ± SEM with 2-tailed Student *t* tests. Significance is represented by *p < 0.05, **p < 0.01, ns indicates not significant. Abbreviations: PCLS, precision-cut liver slices; RT-PCR, reverse transcription polymerase chain reaction.

Next, we examined whether the fibrotic gene expression changes in murine cirrhotic PCLS were also observed in PCLS from human cirrhosis. The human liver samples were confirmed to be cirrhotic (Supplemental Figures S9A–D, http://links.lww.com/HC9/B65). Erlotinib treatment for 72 hours of PCLS from human cirrhotic livers did not change liver morphology (Supplemental Figure S9E, http://links.lww.com/HC9/B65) but significantly inhibited the expression of the profibrogenic genes *COL1A1*, *TIMP1*, *IL6*, and *TNFA* (Figure 7A). No significant effect was observed on the expression of *Tgfb1* and *Acta2*. No significant difference in collagen accumulation was observed after erlotinib treatment (Figures 7B, C). Analysis using PCLS with heatmap demonstrated that the effects of erlotinib as an antifibrotic therapy were similar in murine and human cirrhosis samples (Figure 7D).

## DISCUSSION

While recent advancements in treating viral hepatitis and fatty liver disease have been made, no treatments have been approved for liver fibrosis. New strategies to investigate antifibrotic therapies for cirrhosis are therefore urgently needed. Compared with 2-dimensional in vitro cell lines, PCLS are a better representation of the in vivo environment because they retain the structure and cellular composition of the liver.^7^,^8^ PCLS, therefore, have the capacity to better capture the complex multicellular pathways involved in liver injury and the progression of cirrhosis. Compared to in vivo models, PCLS offer faster readouts, reduced animal numbers, and the ability to run multiple tests on highly similar samples by using serial sections.

In this study, we demonstrated that PCLS are a reliable ex vivo model to evaluate antifibrotic therapies across 4 established murine models of cirrhosis (CDAHFD, TAA, DEN, and CCl_4_), using erlotinib as an example drug. Expression analysis of PCLS after erlotinib treatment showed suppression in a variety of profibrogenic genes. We demonstrated the responses to antifibrotic interventions can be detected and quantified with PCLS at the molecular level. As expected, the morphology of PCLS after erlotinib treatment did not significantly change. This is likely because the short time in culture (72 h) does not provide enough time for significant remodeling to occur.^7^ Our previous *in vivo* experiments showing reduced collagen accumulation in DEN-induced cirrhosis in rats or CCl_4_-induced cirrhosis in mice used longer-term erlotinib treatment for weeks.^23^ Our current study also confirmed that the viability of PCLS from cirrhotic liver stabilized during 72 hours in culture.

We directly compared PCLS with in vivo experiments using the same method of inducing cirrhosis, and showed that many of the acute responses to antifibrotic therapies in PCLS were consistent with in vivo results. Erlotinib treatment of PCLS from DEN-induced cirrhotic rats suppressed the expression of profibrogenic genes, which was consistent with the impact of erlotinib on these genes in vivo. Similar results were demonstrated with the CCl_4_ mouse model.

HSCs expressing *Acta2* are a small minority population within PCLS.^43^ We also compared HSCs in PCLS to common in vitro HSCs. TGF-β1–activated LX2 and TWNT4 cell lines are well-established models to evaluate antifibrotic therapies in vitro.^42^,^44^ Here, we demonstrated that erlotinib treatment significantly suppressed expression of the TGF-β1–induced profibrogenic gene *Acta2* in HSCs in normal liver PCLS. Erlotinib also significantly inhibited the expression of TGF-β1–activated *ACTA2* in LX2 and TWNT4 cells. As other profibrogenic genes, including *Il6*, *Tgfb1*, *Timp1*, and *Col1a1*, are expressed by hepatocytes, HSCs, and other types of liver cells,^45^,^46^,^47^,^48^ the decreased expression of these genes in PCLS after erlotinib treatment might come from all these liver cells. However, erlotinib only significantly inhibited the expression of TGF-β1–activated *COL1A1* in LX2 and TWNT4 cells. *IL6*, *TIMP1*, and *TGFB1* were not reduced after erlotinib treatment, which might also indicate a lower sensitivity for cell lines to respond to certain antifibrotic therapies.

We then investigated other potential mechanisms contributing to these molecular observations of fibrogenesis in PCLS. Matrix metalloproteinases (MMPs) are a family of enzymes that regulate the degradation of extracellular matrix proteins. MMP13 induces inflammation, cytokine secretion, and myofibroblast activation to promote fibrosis. On the contrary, MMP2 and MMP8 induce collagen clearance, and MMP3 inhibits myofibroblast activation to foster an antifibrotic effect. In addition, lack of MMP9 results in reduced fibrosis, and tissue inhibitors of metalloproteinases inhibit the MMPs.^49^ Regulatory pathways on fibrosis generation and degradation^22^,^49^ were significantly altered to exert the antifibrotic therapy of erlotinib in PCLS. After erlotinib treatment, expression of antifibrotic pathways, including *Mmp2*, *Mmp3*, and *Mmp8*, were enhanced in PCLS from CDAHFD-induced cirrhosis, while expression of profibrotic pathways, including *Mmp9* and *Mmp13*, were hampered. *Timp1* expression was also reduced after erlotinib treatment.

We then confirmed the antifibrotic effects of erlotinib using PCLS from human cirrhosis livers. Notably, the fibrotic gene expression changes observed in murine cirrhotic PCLS were also seen in PCLS from human cirrhosis, which addressed the prospect of further assessing the effect of erlotinib in in vivo human cirrhotic liver. The majority of PCLS research to date has focused on murine samples,^8^,^10^,^11^,^13^,^14^,^15^,^16^,^17^,^18^,^19^ and only a small fraction of studies have used human tissue.^12^,^20^ PCLS from human samples may better predict the in vivo response to an antifibrotic therapy in the clinic and also opens up the possibility of using a liver biopsy sample to test an individual patient’s response to a variety of drugs. Human PCLS may also be used as an experimental platform to expedite basic research and drug development by directly testing the effects of drugs in clinical cirrhosis samples rather than animal models.

It should be noted that the culture of PCLS induces a spontaneous fibrogenic reaction.^17^ This unintended activation of fibrogenic pathways during the culture process can potentially limit the effectiveness and applicability of PCLS in screening and testing antifibrotic compounds. This limitation when using PCLS models to ensure accurate evaluation of the antifibrotic properties of new therapeutic agents needs to be further considered.

In summary, the responses to antifibrotic interventions can be detected and quantified with PCLS at the molecular level. PCLS accurately captures the changes in expression that occur in vitro and in vivo during treatment with an antifibrotic therapy. These types of PCLS characterizations were also observed in PCLS from human cirrhosis. PCLS reduces animal numbers, in alignment with the principles of 3Rs,^9^ by enabling many slices to be collected from 1 animal. PCLS is a promising platform for the future development of antifibrotic therapies for cirrhosis.

## Supplementary Material

## Data Availability

All raw data used in the study are available from the corresponding authors upon reasonable request. Kenneth K. Tanabe and Yongtao Wang designed the experiments. Yongtao Wang, Ben Leaker, and Guoliang Qiao conducted the experiments and performed data analysis. Yongtao Wang wrote the manuscript, and Kenneth K. Tanabe and Ben Leaker improved the manuscript. Mozhdeh Sojoodi, Ibrahim Ragab Eissa, Eliana T. Epstein, Jonathan Eddy, Oizoshimoshiofu Dimowo, Georg M. Lauer, Motaz Qadan, Michael Lanuti, Raymond T. Chung, and Bryan C. Fuchs contributed to new reagents or analytical tools.
