# Supplementary material for: Precision-cut liver slices as an ex vivo model to evaluate antifibrotic therapies for liver fibrosis and cirrhosis
Source: Hepatol Commun. 2024 Oct 24;8(11):e0558. doi: 10.1097/HC9.0000000000000558 (PMC11512631; doi:10.1097/HC9.0000000000000558)
Supplement: Supplementary file 1 [file hc9-8-e0558-s001.docx]

**Precision-Cut Liver Slices as an *ex vivo* model to evaluate antifibrotic therapies for liver fibrosis and cirrhosis**

Yongtao Wang^1,2,*^, Ben Leaker^3,4,*^, Guoliang Qiao^1,*^, Mozhdeh Sojoodi^1^, Ibrahim Ragab Eissa^1^, Eliana T. Epstein^2^, Jonathan Eddy^2^, Oizoshimoshiofu Dimowo^2^, Georg M. Lauer^2^, Motaz Qadan^1^, Michael Lanuti^5^, Raymond T. Chung^2^, Bryan C. Fuchs^1^, Kenneth K. Tanabe^1^

**Inventory of Supplementary Information**

The supplementary Information contains 9 supplementary figures and corresponding legends, and 2 supplementary tables.

**Supplementary Figure 1**

**
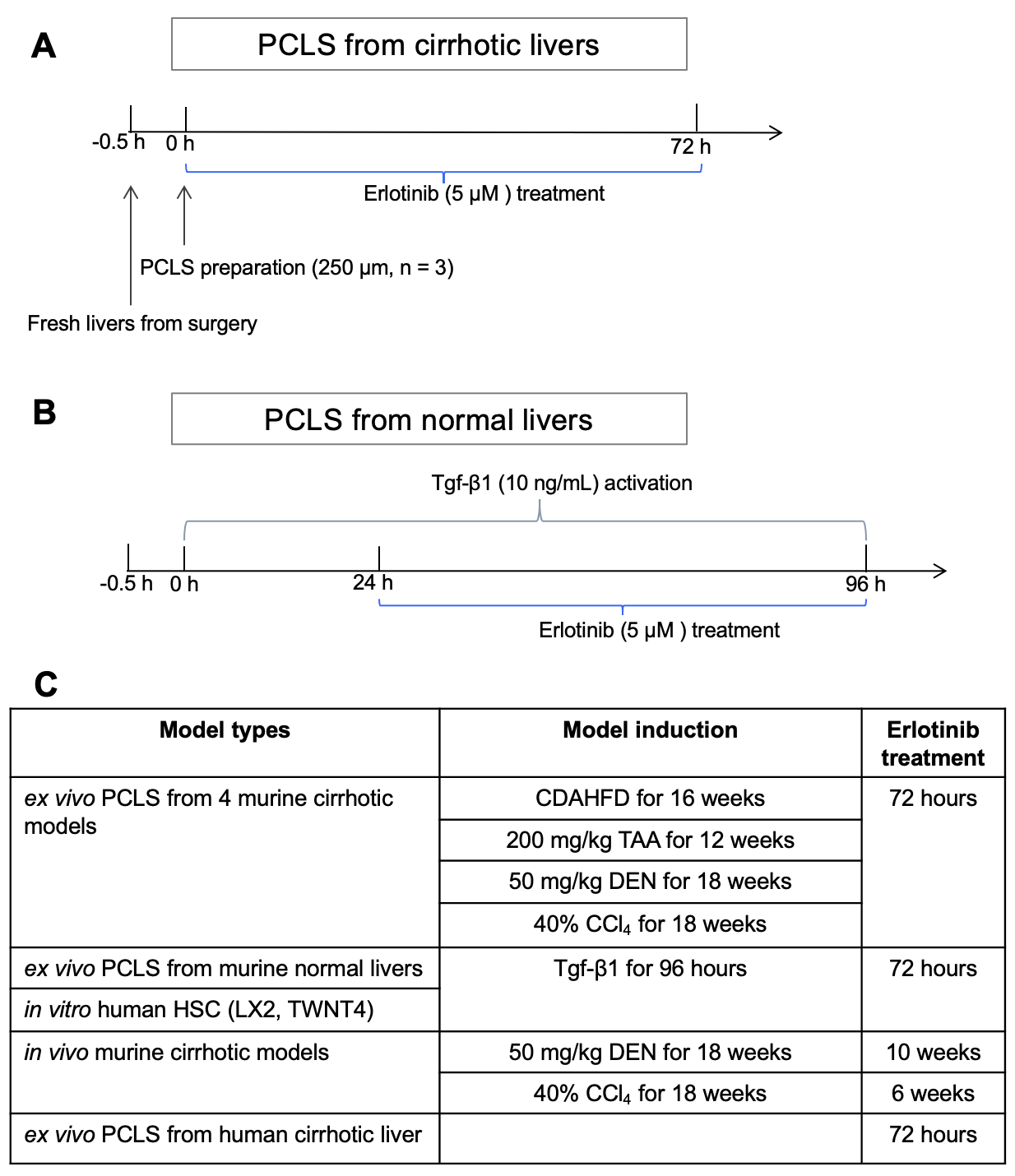
**

**Supplementary Figure 2**

**
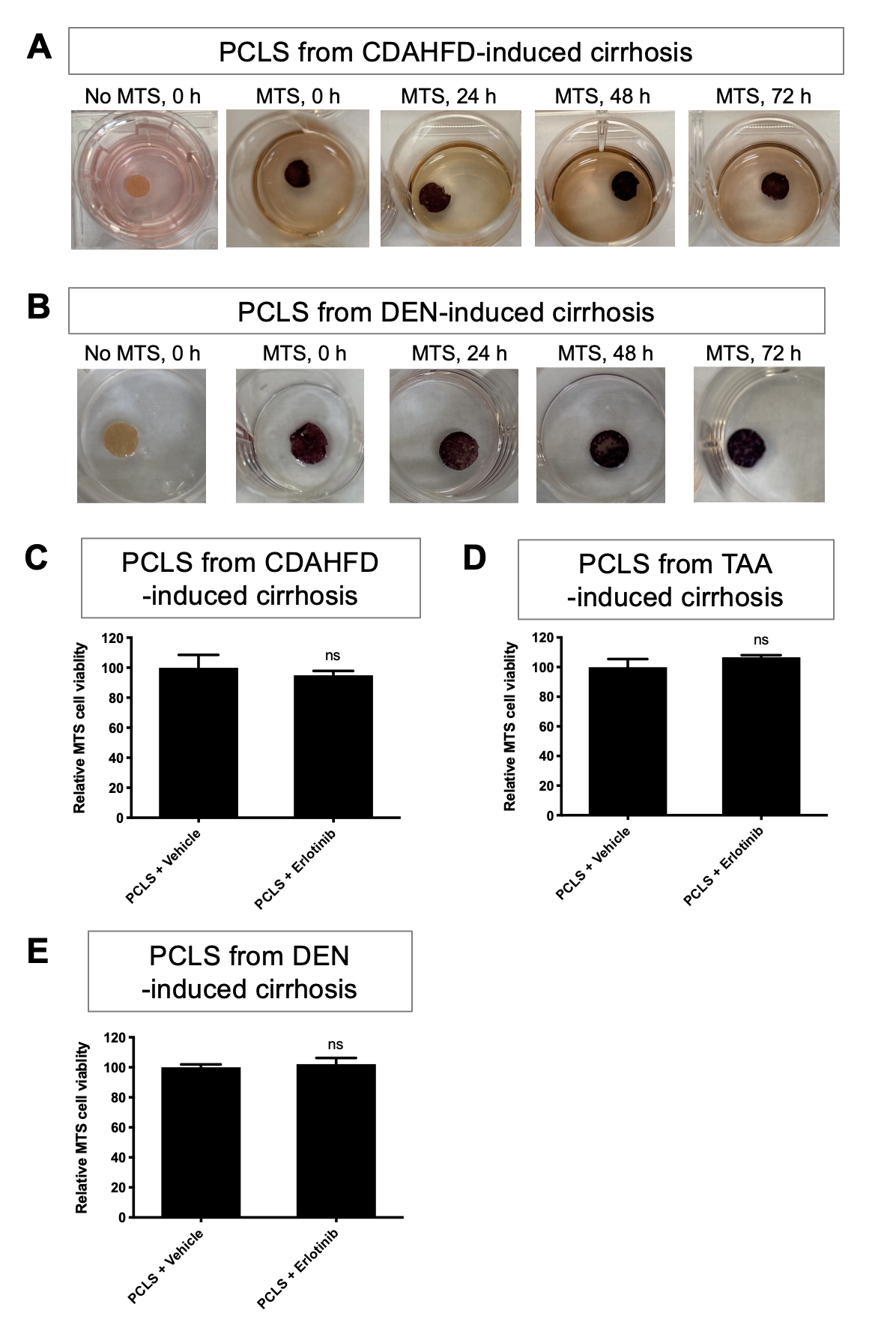
**

**Supplementary Figure 3**

**
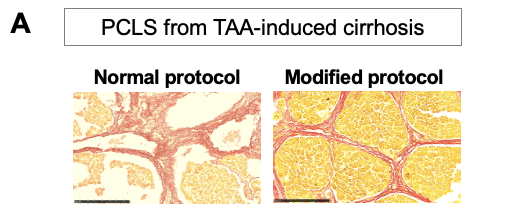
**

**Supplementary Figure 4**

**
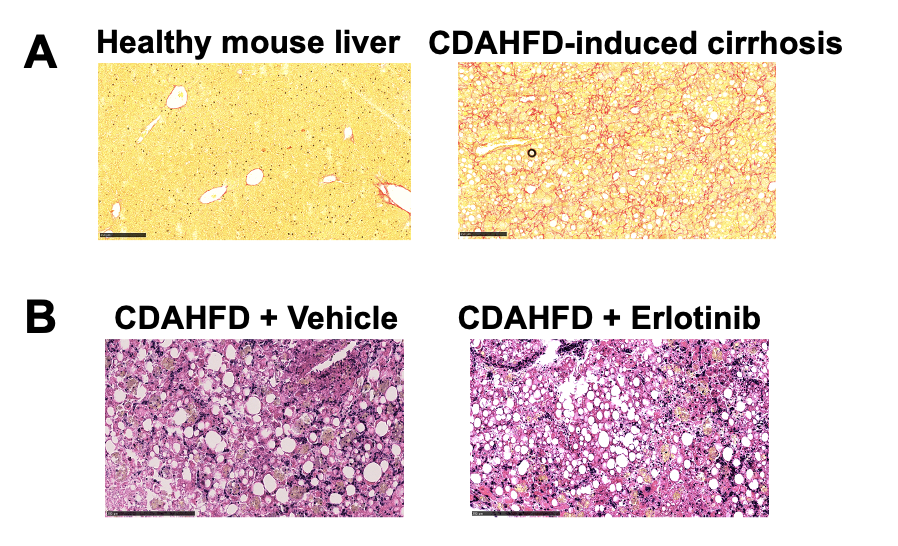
**

**Supplementary Figure 5**

**
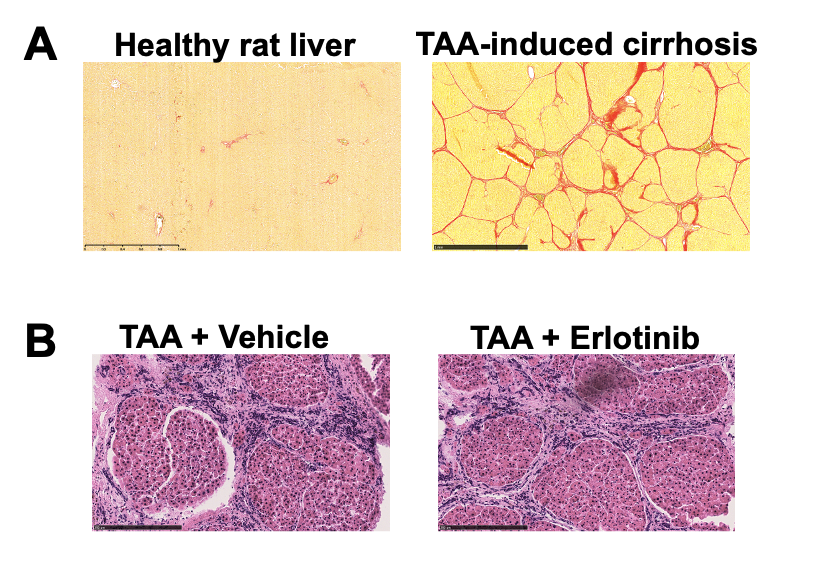
**

**Supplementary Figure 6**

**
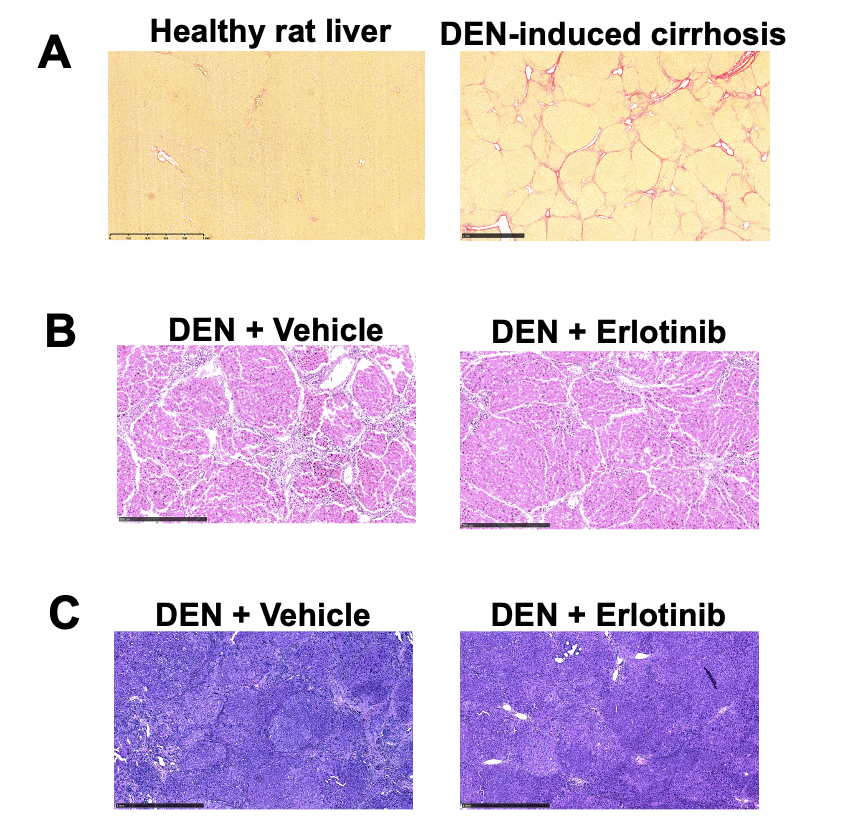
**

**Supplementary Figure 7**

**
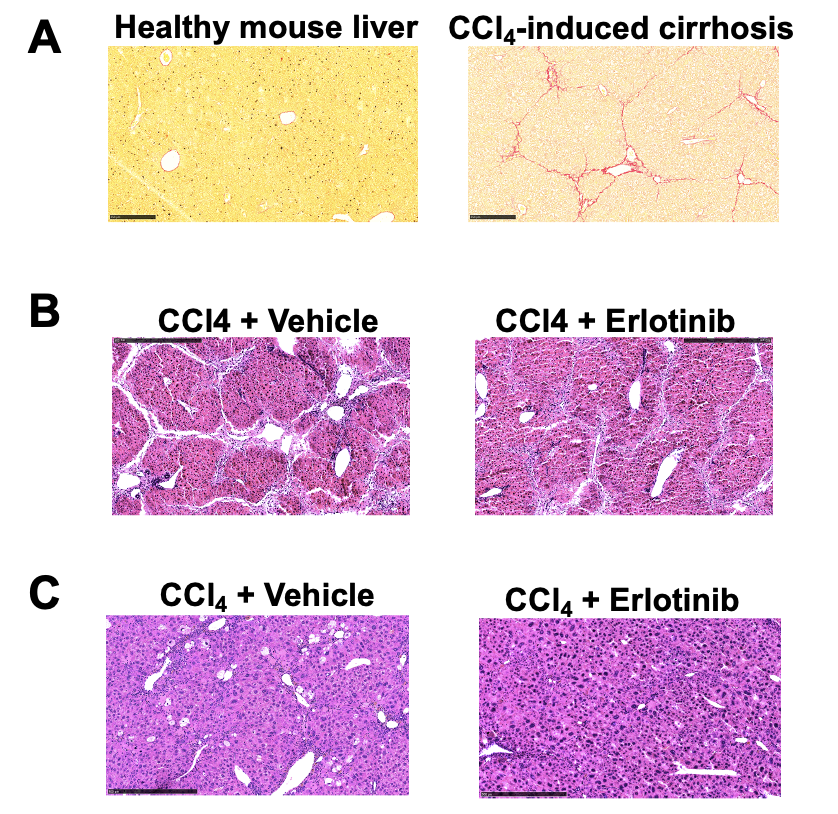
**

**Supplementary Figure 8**

**
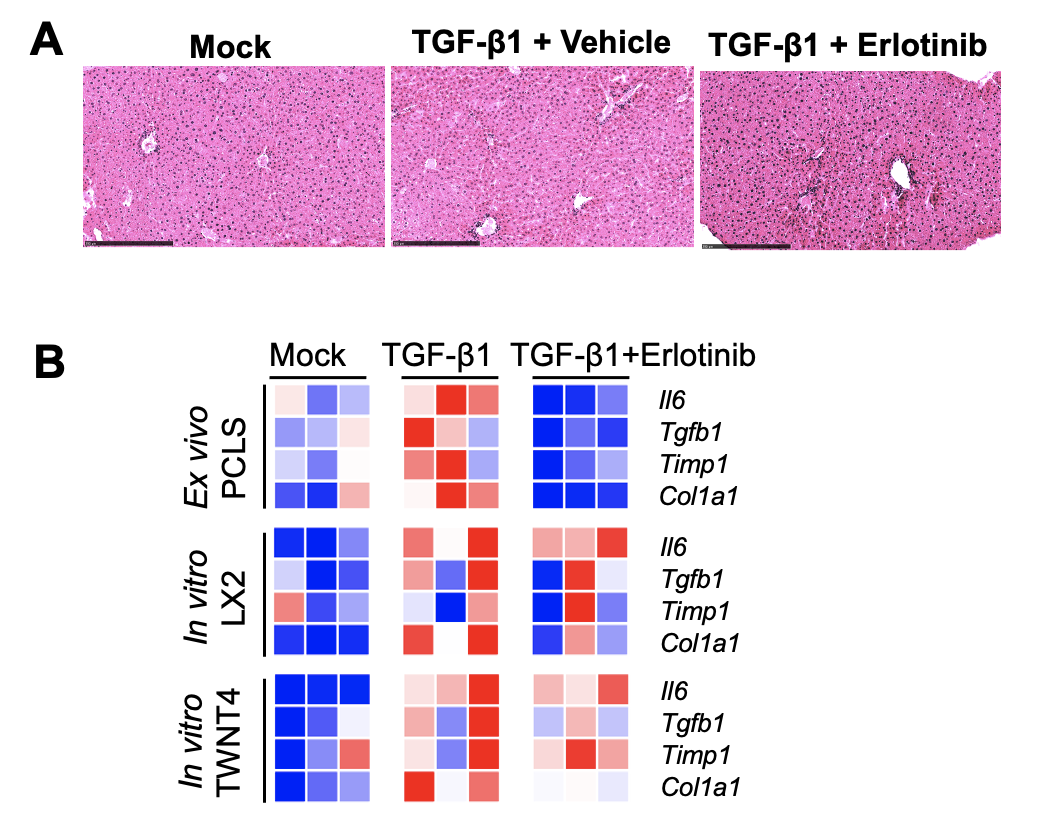
**

**Supplementary Figure 9**

**
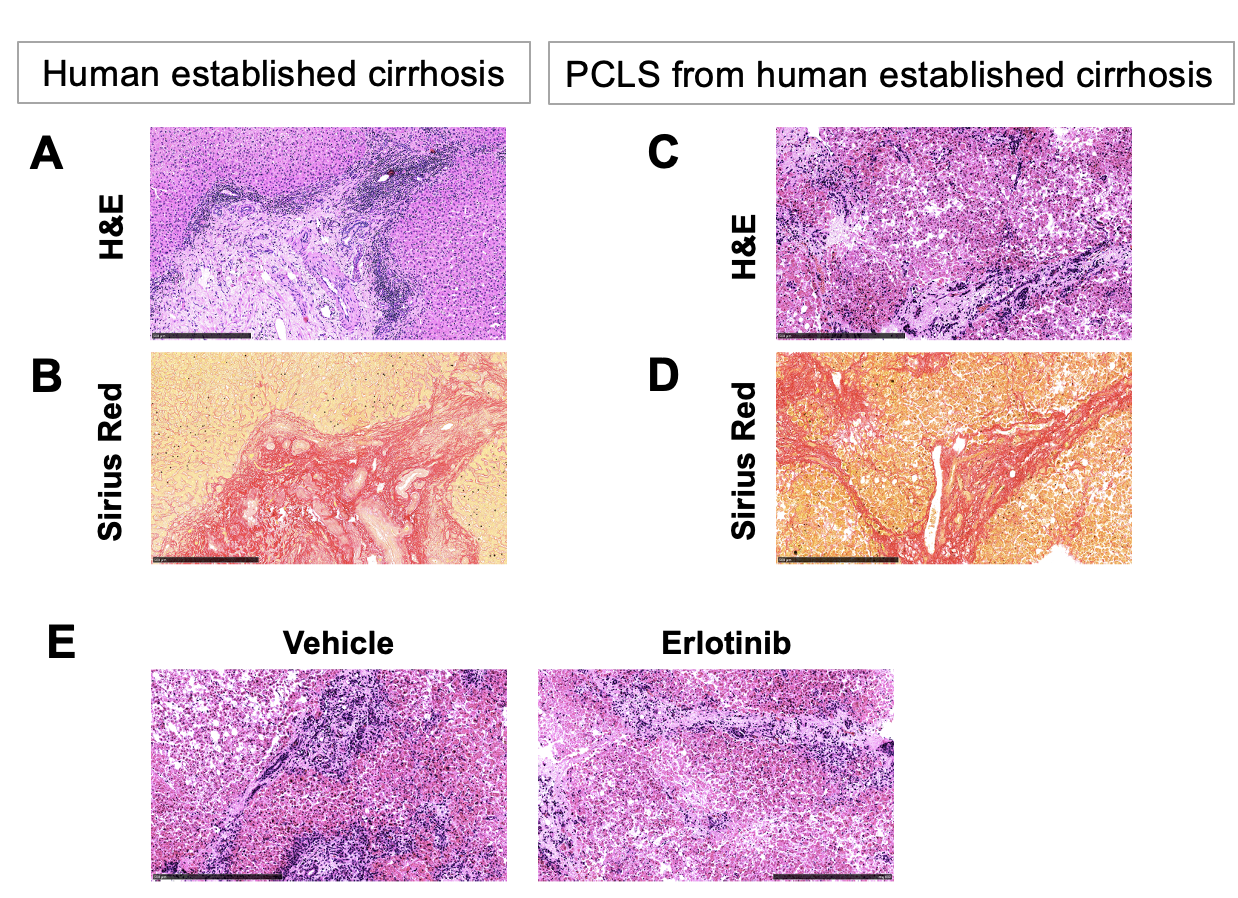
**

**Legends for Supplementary Figures**

**Supplementary Figure 1. Schedule of PCLS preparation and erlotinib treatment.**

Workflow of PCLS preparation and erlotinib treatment for (A) cirrhotic and (B) normal livers. (C) Summary table of PCLS preparation and erlotinib treatment.

**Supplementary Figure 2.** **Viability evaluation in PCLS**

(A) Representative images of PCLS from CDAHFD-induced cirrhosis rats on MTS assays, the cells alive were stained with purple color. (B) Representative images of PCLS from DEN-induced cirrhosis rats on MTS viability assays. MTS assays in PCLS from (C) CDAHFD, (D) TAA and (E) DEN-induced rat cirrhosis after erlotinib treatment. These experiments were repeated three times (n = 3). All values were expressed as the mean ± S.E.M with two-tailed Student’s *t* tests, ns indicates not significant.

**Supplementary Figure 3.** **Improvement of PCLS processing.**

(A) Comparison of standard and modified protocols to proceed PCLS from TAA-induced rat cirrhosis.

**Supplementary Figure 4.** **CDAHFD-induced murine cirrhosis.**

(A) Confirmation of established CDAHFD-induced murine cirrhosis. (B) Representative images for H&E staining in PCLS from CDAHFD-induced mouse cirrhosis.

**Supplementary Figure 5.** **TAA-induced murine cirrhosis.**

(A) Confirmation of established TAA-induced murine cirrhosis. (B) Representative images for H&E staining in PCLS from TAA-induced rat cirrhosis.

**Supplementary Figure 6.** **DEN-induced murine cirrhosis.**

(A) Confirmation of established DEN-induced murine cirrhosis. (B) Representative images for H&E staining in PCLS from DEN-induced rat cirrhosis. (C) Representative images for H&E staining of the samples from *in vivo* DEN-induced rat cirrhotic models.

**Supplementary Figure 7. CCl_4_-induced murine cirrhosis.**

(A) Confirmation of established CCl_4_-induced murine cirrhosis. (B) Representative images for H&E staining in PCLS from CCl_4_-induced mouse cirrhosis**.** (C) Representative images for H&E staining of the samples from *in vivo* CCl_4_-induced mouse cirrhotic models.

**Supplementary Figure 8.** **PCLS from healthy mice livers.**

(A) Representative images for H&E staining of PCLS from male healthy C57Bl/6 mice livers. (B) Heatmap of profibrogenic gene expression for corresponding models.

**Supplementary Figure 9. PCLS from human cirrhosis.**

Confirmation of human established cirrhosis with (A) H&E and (B) Sirius red staining. (C) H&E and (D) Sirius red staining of PCLS from human established cirrhosis. (E) Representative images for H&E staining of human cirrhotic PCLS.

**Supplementary Tables**

**Supplementary Table 1. Modified *Ishak* Scoring**^1^

| Score | Description |
| --- | --- |
| **0** | No fibrosis |
| **1** | Fibrous expansion of some or most portal areas, with or without short fibrous septa |
| **2** | Fibrous expansion of most portal areas, with occasional portal to portal bridging, or with marked portal to portal bridging as well as portal areas to central bridging |
| **3** | Marked bridging with occasional nodules |
| **4** | Probable to definite cirrhosis |

**Supplementary Table 2. Oligonucleotides**

| Gene Symbol | Species Specificity | Company | Assay ID |
| --- | --- | --- | --- |
| *18S* | Human | Thermo fisher | Hs03003631_g1 |
| *IL6* | Human | Thermo fisher | Hs00174131_m1 |
| *ACTA2* | Human | Thermo fisher | Hs00426835_g1 |
| *TIMP1* | Human | Thermo fisher | Hs01092512_g1 |
| *COL1A1* | Human | Thermo fisher | Hs0016004_m1 |
| *TNFA* | Human | Thermo fisher | Hs00174128_m1 |
| *TGFB1* | Human | Thermo fisher | Hs00998133_m1 |
| *Il6* | Rat | Thermo fisher | Rn01410330_m1 |
| *Acta2* | Rat | Thermo fisher | [Rn01759928_g1](https://www.thermofisher.com/taqman-gene-expression/product/Rn01759928_g1?CID=&ICID=&subtype=) |
| *Timp1* | Rat | Thermo fisher | Rn01430873_g1 |
| *Col1a1* | Rat | Thermo fisher | Rn01463848_m1 |
| *Tgfb1* | Rat | Thermo fisher | Rn00572010_m1 |
| *Il6* | Mouse | Thermo fisher | Mm00446190_m1 |
| *Acta2* | Mouse | Thermo fisher | Mm00725412_s1 |
| *Timp1* | Mouse | Thermo fisher | Mm01341361_m1 |
| *Col1a1* | Mouse | Thermo fisher | Mm00801666_g1 |
| *Tgfb1* | Mouse | Thermo fisher | Mm01178820_m1 |
| *Mmp2* | Mouse | Thermo fisher | [Mm00439498_m1](https://www.thermofisher.com/taqman-gene-expression/product/Mm00439498_m1?CID=&ICID=&subtype=) |
| *Mmp3* | Mouse | Thermo fisher | Mm00440295_m1 |
| *Mmp8* | Mouse | Thermo fisher | Mm00439509_m1 |
| *Mmp9* | Mouse | Thermo fisher | Mm00442991_m1 |
| *Mmp13* | Mouse | Thermo fisher | Mm00439491_m1 |
| *Timp2* | Mouse | Thermo fisher | [Mm00441825_m1](https://www.thermofisher.com/taqman-gene-expression/product/Mm00441825_m1?CID=&ICID=&subtype=) |
| *Cd68* | Mouse | Thermo fisher | Mm03047343_m1 |
| *Ccl2* | Mouse | Thermo fisher | [Mm00441242_m1](https://www.thermofisher.com/taqman-gene-expression/product/Mm00441242_m1?CID=&ICID=&subtype=) |
| *Ccr5* | Mouse | Thermo fisher | [Mm01963251_s1](https://www.thermofisher.com/taqman-gene-expression/product/Mm01963251_s1?CID=&ICID=&subtype=) |
| *Cxcl2* | Mouse | Thermo fisher | [Mm00436450_m1](https://www.thermofisher.com/taqman-gene-expression/product/Mm00436450_m1?CID=&ICID=&subtype=) |
| *Cxcr4* | Mouse | Thermo fisher | [Mm01996749_s1](https://www.thermofisher.com/taqman-gene-expression/product/Mm01996749_s1?CID=&ICID=&subtype=) |
| *Ctgf* | Mouse | Thermo fisher | [Mm01192933_g1](https://www.thermofisher.com/taqman-gene-expression/product/Mm01192933_g1?CID=&ICID=&subtype=) |
| *Pdgfrb* | Mouse | Thermo fisher | [Mm00435553_m1](https://www.thermofisher.com/taqman-gene-expression/product/Mm00435553_m1?CID=&ICID=&subtype=) |

**References**

1. Ishak, K. G., Chronic hepatitis: morphology and nomenclature. *Mod Pathol* **1994,** *7* (6), 690-713.
